# Exploring the Role of Novel Medical Therapies for Aggressive Pituitary Tumors: A Review of the Literature—“Are We There Yet?”

**DOI:** 10.3390/cancers12020308

**Published:** 2020-01-28

**Authors:** Lydia S. Lamb, Hao-Wen Sim, Ann I. McCormack

**Affiliations:** 1 Department of Endocrinology, St Vincent’s Hospital, Sydney, NSW 2010, Australia; l.lamb@garvan.org.au; 2 Garvan Institute of Medical Research, Sydney, NSW 2010, Australia; hao-wen.sim@svha.org.au; 3 St Vincent’s Clinical School, University of New South Wales, Sydney, NSW 2010, Australia; 4 Kinghorn Cancer Centre, Sydney, NSW 2010, Australia

**Keywords:** aggressive pituitary tumors, pituitary carcinoma, molecular profiling, targeted therapy, immunotherapy

## Abstract

Aggressive pituitary tumors account for up to 10% of pituitary tumors and are characterized by resistance to medical treatment and multiple recurrences despite standard therapies, including surgery, radiotherapy, and chemotherapy. They are associated with increased morbidity and mortality, particularly pituitary carcinomas, which have mortality rates of up to 66% at 1 year after diagnosis. Novel targeted therapies under investigation include mammalian target of rapamycin (mTOR), tyrosine kinase, and vascular endothelial growth factor (VEGF) inhibitors. More recently, immune checkpoint inhibitors have been proposed as a potential treatment option for pituitary tumors. An increased understanding of the molecular pathogenesis of aggressive pituitary tumors is required to identify potential biomarkers and therapeutic targets. This review discusses novel approaches to the management of aggressive pituitary tumors and the role of molecular profiling.

## 1. Introduction

Pituitary tumors account for approximately 10% to 15% of all intracranial tumors [1], with a prevalence of clinically significant pituitary tumors reported as 80 to 100 cases per 100,000 [2]. The majority are benign and slow growing; however, up to 10% have a more aggressive clinical course [3]. The definition of aggressive pituitary tumors (APTs) has been controversial. The previous edition of the World Health Organisation (WHO) Classification of Tumors of the Pituitary Gland (2004) classified three types of pituitary tumors, including typical adenoma, atypical adenoma, and pituitary carcinoma. Atypical adenomas were defined as tumors with histological features suggestive of aggressive clinical behavior, including an elevated mitotic index, proliferation marker Ki-67 labeling index >3%, and overexpression of tumour protein p53 by immunohistochemistry [4]. In the most recent WHO classification in 2017, the term atypical adenoma is no longer recommended due to a lack of evidence for its prognostic value [5,6,7]. It has been more recently proposed that APT be defined clinically as invasive tumors displaying an unusually rapid tumor growth rate or tumor growth despite optimal standard therapies, including surgery, radiotherapy, and chemotherapy [1,2,3]. The new WHO classification describes pituitary tumor subtypes along with histological markers, such as Ki67 > 3%, that may suggest a potential for aggressive clinical behavior [5]. Pituitary carcinomas (PCs), defined by the presence of craniospinal or systemic metastases, are very rare, comprising just 0.2% of all pituitary tumors [1,5]. The mortality rates of PCs are known to be high, historically up to 66% at 1 year following diagnosis [8,9]. Recently, it has been demonstrated that the mortality associated with APTs approaches that of PCs, with mortality rates for APT of 28% and PC 42.5% at a median duration of 11 years following pituitary tumor diagnosis [3]. Temozolomide is the first chemotherapeutic agent to show activity against APTs and PCs and is now recommended as first-line chemotherapy [2,10]. Overall, temozolomide treatment is associated with a 37% radiological response rate in APTs and PCs, with demonstrated increased survival rates among responders [3,11]. However, 30% of patients demonstrate progression despite temozolomide treatment, and of those that respond, subsequent tumor progression is common after cessation of temozolomide [3]. Thus, there is an urgent need to identify alternative effective treatments to help manage this challenging condition.

Modern day medical oncology has seen a transition towards precision medicine and the use of targeted therapies in the management of cancer. Advances in tumor molecular profiling and increased use of next generation sequencing technology in clinical practice have facilitated the discovery of molecular drivers of cancer and development of treatments to target specific molecular signatures [12,13]. In the management of APTs and PCs, the use of novel targeted therapies has emerged somewhat incidentally on the back of successful application in other cancer types. In addition, there may be a role for immune checkpoint inhibitor therapies, which have revolutionized oncology practice and are now used across multiple cancer types. However, further understanding of the molecular pathogenesis of pituitary tumors will help inform the applicability of such novel therapies.

Herein, we will review the role of novel targeted therapies and initial experience with immunotherapy in the management of APT and PC and propose a focus on the development of molecular profiling of these tumors to help rationalize treatment modalities.

## 2. Targeted Therapies

**Figure 1:**
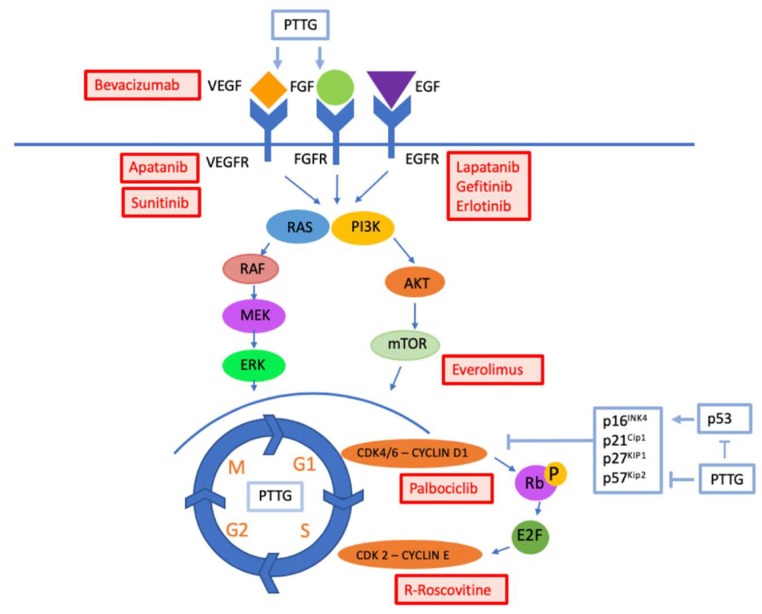
Growth factors, such as vascular endothelial growth factor (VEGF), epidermal growth factor (EGF) and fibroblast growth factor (FGF) bind receptor tyrosine kinases (RTKs) on the cell surface. This initiates intracellular signaling via the P13K/Akt/mTOR and Raf/Mek/ERK pathways, which promotes cell cycle progression within the cell nucleus. Cell cycle progression is activated by cyclins binding to cyclin-dependent kinases and inhibited by a number of proteins, including p16, p21, p27, p57, as well as p53, which acts by upregulating p21. Pituitary tumour transforming gene (PTTG) is an oncogene involved in multiple cellular processes, including induction of growth factors VEGF and FGF; downregulation of cell cycle inhibitors, such as p21; and modulation of cell cycle progression. Targeted therapies have been developed, which interrupt the cellular signaling pathway at different points and act to downregulate cellular differentiation and proliferation in cancer.

Growth factors and their receptors, intracellular signaling pathways, and proteins involved in cell cycle regulation are all known to have important roles in pituitary tumorigenesis and thus are potential targets for therapy (Figure 1, Table 1 and Table 2).

## 3. Growth Factor-Targeted Therapies

### 3.1. Inhibition of Vascular Endothelial Growth Factor (VEGF)

Vascular endothelial growth factor (VEGF) plays a key role in tumorigenesis by promoting angiogenesis and vascular permeability as well as modulating the tumor immune microenvironment [26,27]. VEGF contributes to the pro-tumor immunosuppressive microenvironment by inhibiting dendritic cell maturation, accumulating myeloid-derived suppressor cells, and inducing T regulatory cells (T reg), as well as increasing the expression of inhibitory receptors on cytotoxic CD8+ T cells, including programmed cell death protein 1 (PD-1), cytotoxic T lymphocyte associated protein 4 (CTLA-4), T cell immunoglobin and mucin domain containing protein 3 (TIM-3), and lymphocyte activation gene 3 (LAG-3) [28,29]. VEGF is upregulated in many human tumors [27] by common genetic events, which lead to malignant transformation, such as a loss of tumor suppressor genes and activation of oncogenes. Hypoxia, a common neoplastic characteristic, is also a driver of VEGF expression and upregulation of VEGF tyrosine kinase receptors (VEGFRs) VEGFR-1 and VEGFR-2 [30]. In turn, VEGFR-1 and to a greater extent VEGFR-2 are major transducers of important oncogenic downstream signaling pathways, such as phosphoinositide 3-kinase (PI3K)/Akt and mitogen activated protein kinase (MAPK) pathways [26].

Targeted therapy with antibodies directed at VEGF and VEGFR are now used in the treatment of many cancers [26]. Bevacizumab, a recombinant monoclonal antibody given intravenously, blocks VEGFA and inhibits the interaction between VEGF and VEGFR. It prolongs progression-free survival in metastatic colorectal cancer, cervical cancers, non-small cell lung, ovarian cancer, mesothelioma, and metastatic renal cell carcinoma [31]. However, in some cancers, bevacizumab has not been demonstrated to increase survival and the explanation for this lack of improvement remains unknown [31]. Apatinib, an oral therapy, inhibits VEGFR2 predominantly but has mild inhibitory effects on other tyrosine kinase receptors, such as c-Kit and c-Src [32]. Clinical trials have demonstrated improved progression-free and overall survival with apatinib treatment in gastric, breast, and lung cancer [32].

The mechanism of action of anti-VEGF therapy is multifactorial and includes inhibition of angiogenesis, induction of epithelial cell apoptosis, direct effect on tumor cells expressing VEGF receptors, modulation of the immune system with enhanced cytotoxic T cell and dendritic cell activity, and reduced activity and accumulation of immunosuppressive T reg cells, tumor-associated macrophages, and myeloid-derived suppressor cells [29,33].

Markers of tumor angiogenesis, such as VEGF expression, have been found to be higher in APT compared with non-aggressive pituitary adenomas [34,35,36,37]. VEGF expression and secretion is reduced by octreotide in growth hormone (GH)-secreting adenomas and pasireotide in non-functioning (NF) adenomas in vitro, suggesting that somatostatin analogues may inhibit pituitary tumor cell viability by inhibiting the action of VEGF [38,39]. In mice with and without multiple endocrine neoplasia 1 (MEN1), anti-VEGF therapy reduces tumor size, prolactin secretion, and vascularity in prolactin-secreting adenomas [40,41,42] as well as normalizing blood vessels and reducing intra-tumoral hemorrhage [42].

Thirteen cases have been described of APT or PC targeted with anti-VEGF or anti-VEGFR therapy. Nine cases have been reported to respond to treatment with the VEGF antibody bevacizumab. Four of these patients were treated concurrently with temozolomide and bevacizumab with or without radiotherapy, including one receiving a second course of temozolomide and five cases commenced bevacizumab following progressive disease on temozolomide, including one case in combination with pasireotide [3,14,15,16,17,18]. One case has been reported of an aggressive GH-secreting adenoma responding to treatment with a combination of temozolomide and the VEGFR-2 inhibitor apatinib, with normalization of GH and a >90% reduction in the tumor size [19]. Two cases have been reported of progressive disease on bevacizumab following previous chemotherapy. In addition, one case with progressive disease on the VEGFR inhibitor sunitinib is reported [3]. Based on these outcomes, VEGF-targeted therapy is a promising therapeutic option; however, further investigation is required to identify predictors of response to treatment and guide patient selection.

### 3.2. Inhibition of Epidermal Growth Factor Receptors (EGFRs)

The epidermal growth factor receptor (EGFR) family comprises EGFR (ErbB1/HER1), ErbB2 (ErbB2/HER2/Neu), Erb B3 (ErbB3/HER3), and Erb B4 (ErbB4/HER4), a group of membrane receptor tyrosine kinases that promote cell proliferation and suppress apoptosis [43]. Mutations to and overexpression of EGFR commonly occurs in cancer and leads to the upregulation of the downstream PI3K/Akt/mammalian target of rapamycin (mTOR) and Raf/mitogen-activated protein kinase (Mek)/extracellular signal-regulated kinase (ERK) pathways, which promote cancer cell proliferation [43,44,45]. Increased EGFR expression has been associated with poorer clinical outcome in a number of malignancies, including bladder, breast, lung, and head and neck cancers [45].

Given the role of EGFR and its signaling pathways in oncogenesis, two types of EGFR-targeted therapies have been developed. Humanized monoclonal antibodies (mAbs) against the EGFR extracellular domain block ligand binding and mediate downregulation of EGFR while tyrosine kinase inhibitors (TKIs) bind to the receptor pocket, excluding ATP and thus preventing signal transduction [43,45]. These therapies have been shown to improve survival in EGFR-positive cancers and have been approved for use in non-small-cell lung cancer, metastatic colorectal cancer, head and neck cancer, pancreatic cancer, and breast cancer [43,46]. Predictors of response to EGFR-targeted therapy have been examined. In patients with non-small cell lung cancers, high levels of EGFR protein expression or increased *EGFR* copy numbers derive greater therapeutic benefit from EGFR directed mAbs [47,48]. Mutations in exon 18-21 of *EGFR* cause variable activation of EGFR in the absence of ligand binding, driving tumorigenesis. The majority of these mutations confer sensitivity to EGFR blockade, with rare activating mutations conferring resistance [48]. Tumor analysis for EGFR mutations is therefore routine in clinical practice to predict response to therapy in lung adenocarcinoma [49].

In pituitary tissue, EGFR is expressed at variable levels in normal pituitary as well as functioning and non-functioning pituitary adenomas, with higher expression demonstrated in invasive adenomas and carcinomas [44,50,51]. EGFR overexpression in corticotroph adenomas is associated with downstream activation of the ERK pathway. The expression levels of EGFR were found to correlate with adrenocorticotroph hormone (ACTH) and cortisol levels as well as tumor recurrence [52]. Increased EGFR expression in corticotroph adenomas has been attributed to recently identified somatic mutations in ubiquitin-specific peptidase 8 (USP8), a deubiquitinase enzyme that protects EGFR from degradation. USP8 mutations are located in the 14-3-3 protein binding motif and enhance the proteolytic cleavage and catalytic activity of USP8, resulting in increased EGFR signaling. USP8-mutated tumors have demonstrated higher levels of proopiomelanocortin (POMC), the precursor to ACTH [53,54]. Interestingly, surgical remission rates are higher in patients with USP8-mutated tumors, which may reflect these tumors are smaller and thus may suggest USP8 mutations are not a critical factor in the development of aggressive tumor behavior. There are likely to be other, currently unidentified, factors that may also contribute to EGFR signaling in pituitary tumors. Nevertheless, these findings raise the possibility of EGFR-directed therapy, particularly in corticotroph adenomas.

Gefitinib, a TKI-targeting EGFR, suppresses expression of POMC and ACTH secretion in murine and canine corticotroph adenoma cell cultures, suppresses expression of POMC in human corticotroph adenoma cell cultures, as well as attenuating the growth and ACTH secretion of EGFR-expressing tumors in vivo in mice [55]. In GH3 rat lactosomatotroph pituitary tumor cells transfected with EGFR2, lapatanib, a dual EGFR/ErbB1 and HER2/ErbB2 TKI, has been shown to suppress prolactin mRNA expression and secretion, and reduce tumor volume to a greater effect than gefitinib in both in vivo rat models and in vitro human prolactinoma cell lines [56].

To date, seven cases have been reported of lapatinib therapy for aggressive prolactinomas, with variable results. Tumor size decreased by 22% in one patient, remained stable in three, and increased in three. Prolactin level decreased in three patients, with a median decrease of 42%, but increased in the other three cases [3,20]. Lapatinib has also been used unsuccessfully in one case of an aggressive silent GH tumor and an aggressive ACTH-secreting adenoma with disease progression [3].

### 3.3. Inhibition of Fibroblast Growth Factor

Fibroblast growth factor (FGF) and their receptors are involved in the regulation of cellular proliferation, survival, migration, and differentiation, and play a key role in angiogenesis. Dysregulation of FGF signaling contributes to oncogenesis by driving cellular proliferation and promoting angiogenesis [57,58]. FGF receptor (FGFR) mutations and amplifications have been implicated in a number of cancers, including multiple myeloma, bladder, cervical, prostate, breast, and gastric cancer [57]. Paradoxically, FGF-2 has been demonstrated to inhibit cellular proliferation and promote apoptosis in certain human breast cancer cell lines as well as being associated with good prognostic indicators when over expressed in some breast cancers [58]. In addition, FGFR-2 expression is associated with decreased tumor progression in some tumors, such as astrocytomas, bladder, prostate, and thyroid carcinomas [58].

In normal pituitary tissue, FGF has been reported to be produced by the folliculostellate cells and regulates GH, prolactin, and TSH secretion [59]. There are four distinct FGFRs (FGFR 1,2,3, and 4); however, normal pituitary tissue expresses the mRNA for only FGFR-1, FGFR-2, and FGFR-3 along with the secretable immunoglobulin-like domain of FGFR-4 [60,61]. In around 60% of pituitary adenomas, an N-terminally truncated FGFR-4 isoform has been identified, which includes a transmembrane and kinase domain not identified in normal pituitary tissue. The expression of pituitary tumor-derived FGFR-4 is increased in macroadenomas compared with microadenomas and correlates with Ki67 and tumor invasiveness [61]. In pituitary adenomas, higher expression of FGF2 and FGFR-1 has been demonstrated in comparison with normal pituitary, with significantly more enhanced expression of FGFR-1 in invasive tumors [62]. In contrast, FGFR-2 has been shown to be downregulated in pituitary adenomas due to FGFR-2 promoter methylation; however, this has not been clinically assessed in relation to tumor invasiveness or aggressiveness [63].

Therapies targeting FGF and their receptors have been proposed in the management of cancer, and FGFR tyrosine kinase inhibitors are currently being investigated in preclinical and clinical trials [64]. The complex involvement of FGF and their receptors in the pathogenesis of pituitary tumors suggests there may be a role for therapies targeting this pathway in the treatment of pituitary tumors, although this has not yet been investigated.

## 4. Targets of Intracellular Signaling Pathways

### 4.1. PI3K/Akt/mTOR and Raf/Mek/ERK Pathways

Under normal circumstances, cellular regulation is under the control of complex intracellular signaling pathways, including the PI3K/Akt/mTOR and Raf/Mek/ERK pathways, which regulate cell cycle proliferation and differentiation, cell survival, protein synthesis, and cellular metabolism [65,66] (Figure 1). Signal transduction is initiated by growth factors activating receptor tyrosine kinases (RTKs). In the PI3K/Akt/mTOR pathway, RTKs activate the signal transduction component lipid kinase, PI3K. This then activates Akt, which phosphorylates several downstream targets, leading to cell proliferation [65,66,67]. In the Raf/Mek/ERK pathway, Ras GTPase stimulation results in Raf phosphorylation and activation of MEK1 and MEK2, which in turn phosphorylate and activate ERK1 and ERK2. ERK signaling targets multiple kinases, phosphatases, transcription factors, and cytoskeletal proteins, resulting in the modulation of genes associated with cell proliferation [66,67]. A major downstream target of both the PI3K/Akt/mTOR and Raf/Mek/ERK pathway is mTOR, which stimulates cell growth. Mutations in these pathways, in mTOR itself and in the mTOR complexes mTORC1 and mTORC2, account for up to 30% of all human cancers [66,68].

Therapies that inhibit mTOR prolong progression-free survival and have been approved for use in multiple cancers, including renal cell carcinoma, neuroendocrine tumors, and advanced breast cancer, with trials ongoing in other malignancies [69,70,71,72]. mTOR inhibitors seem to demonstrate better efficacy in renal cell carcinoma and breast cancer than in other malignancies, which is interesting as they share similar genetic alterations in the mTOR signaling pathway, including mutations to PI3KCA and mutations and deletions of phosphatase and tensin homolog (PTEN). This may suggest a role for genetic and molecular analysis to predict response to treatment [73]. Potential biomarkers of response to mTOR inhibition, including loss of PTEN function, AKT phosphorylation, and PI3K, mTOR, tuberous sclerosis complex (TSC) 1 or 2 mutations [47,69,74]; and those that predict resistance, including overexpression of the apoptosis-inhibitory protein Bcl-2 and KRAS, BRAF, and TSC mutations [47,69]. Validation of these biomarkers in clinical trials is required.

Both the PI3K/Akt/mTOR and Raf/Mek/ERK pathways are upregulated in pituitary tumors and implicated in pituitary tumorigenesis [67]. The *PI3KCA* gene encodes PIK3CA of class IA PI3K and *PI3KCA* gene mutations and amplifications cause increased enzymatic activity of PIK3CA, activation of AKT signaling, and growth factor-independent growth, invasion, and metastasis of cancer cells [75]. Amplifications of the *PI3KCA* gene are observed in invasive and non-invasive pituitary tumors, with a prevalence of 20% to 40% [75,76]. *PI3KCA* mutations are present in pituitary tumors [76] and are particularly associated with invasive tumors, with one study demonstrating somatic mutations in 9% of invasive pituitary tumors but none among the cohort of non-invasive tumors. Furthermore, the presence of a *PI3KCA* mutation was associated with increased recurrence rates [75]. Akt mRNA expression, phosphorylated Akt, and Akt activity are increased in pituitary tumors compared with normal pituitary tissue [21,67,77]. The expression of B-Raf mRNA and phosphorylated MEK1/2 and ERK1/2 are also significantly higher in pituitary adenomas compared to normal pituitaries [67,78]. Interestingly, *H-ras* point mutations have been reported in distant metastatic pituitary tumor deposits of three patients but not in their respective primary pituitary tumors, suggesting that *H-ras* mutations may have a unique role in the development of pituitary metastases [79]. The oncogenic *V600E BRAF* mutation has been identified in 16.5% of corticotroph adenomas but not in other types of pituitary adenomas. In a murine corticotroph cell line, increased phosphorylation of ERK1/2 was demonstrated in cells expressing the *BRAF* mutation, consistent with increased MAPK activity. These cells also exhibited increased levels of *POMC* mRNA [80].

Given the important role of the PI3K/Akt/mTOR and Raf/Mek/ERK pathways in pituitary tumor pathogenesis, a number of agents targeting these pathways have been investigated in the treatment of pituitary adenomas; however, most evidence of activity comes from preclinical studies. Treatment of murine corticotroph pituitary tumor cells with the *BRAF* inhibitor vemurafenib resulted in a greater reduction of ACTH in the cells expressing the *V600E BRAF* mutation compared to tumor cells with wild-type *BRAF*, suggesting a potential role for *BRAF* inhibitor therapy in patients with *BRAF*-mutated tumors [80]. Rapamycin is a lipophilic macrolide that acts to inhibit mTOR, leading to a reduction in cell cycle progression and cell proliferation [68,81]. In rat GH-secreting pituitary tumor GH3 cell lines, rapamycin, its bioavailable analog RAD001 (Everolimus), and PI3K inhibitors have been shown to decrease cell viability and proliferation through inhibition of mTOR activity and interruption of normal cell cycle function [82,83]. These effects appear to occur without elimination of the p70S6K negative feedback loop, which leads to treatment resistance in other cancers. However, in human GH-secreting pituitary adenoma cell lines, everolimus has produced a reduction in cell viability in one study but not in another [82,83]. The dual mTOR/PI3K inhibitor NVP-BEZ235 reduces cell proliferation and promotes cell death in rat non-functioning pituitary adenomas in vitro and in vivo [84], in growth hormone-secreting GH3 cell lines in vitro, and in primary cell cultures of human prolactinomas [85]. The pan-PI3K inhibitor NVP-BKM120 (Buparlisib) has demonstrated no significant effect on GH3 cells in vitro but did reduce the tumor volume, prolactin secretion, and mitotic index in rat models of prolactin tumors in vivo [85]. Synergies may also be effective. The combination of everolimus and cabergoline was shown to inhibit mammosomatrotroph tumor GH3 cell proliferation and prolactin levels in vitro [21]. The combination of dual PI3K/mTOR inhibition and temozolomide has been demonstrated to synergistically inhibit tumor growth and reduce GH/PRL levels in pituitary adenoma cell lines and in a mouse GH3 tumor model [86]. In other cancers, including glioblastoma, melanoma, and renal cell carcinoma, this combination has been effective in overcoming temozolomide resistance [86].

Clinical experience with everolimus in pituitary tumors is limited to case reports with variable outcomes. Two cases have been described with biochemical and radiological response to everolimus [21,22]. Both cases had abnormalities demonstrated in the mTOR signaling pathway, including an STK11 mutation in an ACTH-secreting PC and increased p-Akt expression in an aggressive prolactinoma [21,22]. In contrast, in another case of a recurrent ACTH-secreting PC, there was no response to the combination of everolimus and octreotide [23]. This may have been due to the development of rapamycin resistance due to the feedback of rapamycin on the phosphorylation of Akt, a mechanism that seems to be absent in GH- and prolactin-secreting tumors [23]. Three other cases reported had progressive disease despite treatment with everolimus [3].

### 4.2. Notch and Hedgehog Signaling Pathways

The Notch signaling pathway regulates normal cell proliferation and differentiation and is of relevance in cancer biology, affecting multiple tumor cell types, including cancer stem cells, immune, endothelial, and tumor cells [87]. Agents targeting the Notch signaling pathway are under investigation in the treatment of cancer and response has been demonstrated in phase 1 and 2 clinical trials in colorectal, breast, lung, ovarian and papillary thyroid cancer, anaplastic astrocytoma, sarcoma, glioblastoma multiforme, and melanoma [87,88].

The hedgehog signaling pathway is implicated in the maintenance and homeostasis of stem cells and regulates cell proliferation, survival, and angiogenesis [87]. Aberrations in the hedgehog pathway have been implicated in many cancers, including pancreatic, colon, gastric, lung, breast and prostate cancer, basal cell carcinoma, medulloblastoma rhabdosarcoma, leukemia, and multiple myeloma [89]. Vismodegib, which is an antagonist of the smoothened (SMO) receptor in the hedgehog pathway, increases overall survival in metastatic basal cell carcinoma (BCC) and has been approved for use in patients with metastatic or locally advanced BCC who are not candidates for surgery or radiotherapy. Clinical trials are ongoing to examine the role of Vismodegib and another SMO inhibitor Saridegib in other cancers; however, no benefit has yet been demonstrated in gastric, lung, colorectal, ovarian, pancreatic cancer, myelofibrosis, or chondrosarcoma [87].

The Notch and hedgehog pathway are involved in normal pituitary development and there is evidence for their role in pituitary tumor pathogenesis [90]. The Notch 3 receptor and its ligand Jagged1 are increased in non-functioning pituitary adenomas and prolactinomas compared with normal pituitary [91,92]. The expression of the signaling protein sonic hedgehog (SHH) is downregulated in pituitary adenomas and absent in corticotroph adenomas compared with normal pituitary. Furthermore, administration of SHH in AtT20 corticotroph cells exerted antiproliferative effects whereas administration of a SHH inhibitor increased proliferation in GH3 mammosomatotropinomas [93]. These findings suggest that downregulation of the hedgehog pathway may play a role in the pathogenesis of pituitary adenomas [90]. On the other hand, in primary cell cultures established from pituitary adenomas, exogenous SHH increased secretion of GH, prolactin, and ACTH from somatotropinomas, lactotropinomas, and Cushing’s tumors [93]. Further research is required to understand the role of the Notch and hedgehog signaling pathways in pituitary pathogenesis as they may represent novel targets for therapy in the management of APT and PC, although this has not yet been investigated.

## 5. Cell Cycle-Targeted Therapy

Normal cellular proliferation relies on progression through the cell cycle under the control of cyclin dependent kinases (CDKs), which are modulated by activators (cyclins) and inhibitors (Ink4, and Cip and Kip inhibitors), composed of p21, p27, and p57 [94]. In normal functioning cells, CDK 4 and 6 form a complex with cyclin D1 to initiate phosphorylation of retinoblastoma tumor suppressor protein (Rb), which releases suppression of E2F transcription factors. Activated E2F results in the transcription of genes, including cyclin E, which binds CDK2, forming a complex that promotes cell cycle progression from G1 to S. This process is regulated by p16, a CDK inhibitor, which when activated, inhibits the CDK4/6 and cyclin D1 complex, leading to hypophosphorylated Rb and halting cell cycle progression [95,96]. The tumor suppressor gene *p53* also plays an important role in cell cycle regulation by upregulating the expression of the CDK inhibitor p21 [94]. Cell cycle disruption with unregulated cell cycle progression is a common feature of human cancer [24,94,95,96]. 

Therapies targeting the cell cycle have recently been developed for use in human cancers. In particular, CDK4/6 inhibitors prolong progression-free survival and are now approved for use in estrogen receptor-positive breast cancer [97,98]. Cyclin D1 deficiency has been shown to impair mammary epithelial proliferation [99] while cyclin D1 and CDK4 are necessary for breast cancer development in mice [100] and cyclin D1 is overexpressed in the majority of human breast cancers [101].

In pituitary tumors, reductions in pRb and p16 or increased expression of cyclin D1 are observed in up to 80% of tumors [67,102,103]. In particular, cyclin D1 is overexpressed in aggressive, non-functioning tumors [104] while cyclin E is overexpressed and p27 reduced in Cushing’s disease [104,105]. Mutations in the tumor suppressor gene *TP53* have been identified rarely in corticotroph adenomas and may provide a therapeutic target in the future [106,107,108]. Clinical trials into the restoration of wild-type p53 in other cancer types are ongoing, but this has not been explored in APT. R-roscovitine is a cell cycle inhibitor that acts on cyclin E/CDK2 to cause cell cycle arrest. In mouse ACTH-secreting pituitary cells, treatment with R-roscovitine reduced the cell number and induced cell cycle arrest, demonstrated by decreased cyclin E, increased p27Kip1, p57Kip2, and p21Cip1 expression, as well as reduced Thr821 phosphorylation of Rb [109]. R-roscovitine treatment also induced senescence as evidenced by increased β-gal expression, and caused a reduction in ACTH expression and secretion. In vivo, mice with an induction of corticotroph tumors treated with R-roscovitine demonstrated a reduction in tumor size as well as serum and tumor ACTH expression [109]. Ander et al. reported an interesting case that supports a potential role for cell cycle inhibition in the treatment of pituitary tumors. A 71-year-old female with an incidental pituitary macroadenoma under observation was treated with the CDK4/6 inhibitor palbociclib for metastatic breast cancer, which resulted in significant regression of the pituitary tumor [24].

## 6. Pituitary Tumor-Transforming Gene (PTTG)

Pituitary tumor-transforming gene (PTTG) is an oncogene involved in multiple cellular processes, including cellular proliferation, DNA repair, angiogenesis, and induction of genetic instability [110]. PTTG interferes with normal cell cycle progression via complex mechanisms, including inhibition of sister chromatid separation in anaphase, interaction with the transcription factor Sp1 to mediate S1/G phase transition, interaction with the p53 pathway, and suppression of the activity of CDK inhibitor p21. PTTG is also involved in angiogenesis via induction of FGF and VEGF [110]. Overexpression of PTTG contributes to cellular transformation and tumor development and has been implicated in endocrine and non-endocrine tumors, including those of the pituitary, thyroid, ovary, breast, prostate, lung, esophagus, colon, and central nervous system [110,111]. PTTG is overexpressed in approximately 90% of pituitary adenomas compared with low or no expression in normal pituitary tissue and PTTG has been demonstrated to correlate with Ki67 and tumor invasiveness and aggression [111,112,113].

Targeting PTTG has been explored in prolactinoma, glioma, follicular thyroid, and cervical cancer cell lines as well as in vivo mouse models of lung cancer and hepatoma and demonstrate the therapeutic promise of PTTG inhibition [110]. In rat prolactin- and GH-secreting pituitary tumor cell lines, overexpression of c-terminal-truncated PTTG suppressed prolactin promotor activity, mRNA expression, and hormone levels. In addition, injecting rats with GH3 cells transfected with truncated PTTG resulted in smaller tumors compared with those injected with the control vector [114]. These findings demonstrate how the interruption of PTTG action may have therapeutic potential in the management of prolactinomas.

## 7. Pituitary Tumor Epigenetics

Epigenetics is defined as a process that heritably influences gene expression without genetic change to the underlying gene sequence and occurs via modifications, including methylation of cytosine bases in DNA, modification of histone proteins, and expression of microRNA [115,116]. A number of drugs have been developed for use in various tumors types that reverse DNA methylation and histone modifications through the inhibition of the enzymes responsible for these changes, including DNA methytransferases (DNMTs) and histone deacetylases (HDACs) [117].

In pituitary tumors, a number of epimutations have been identified that affect the transcription of hormone and growth factor receptors, signal transduction pathway molecules, transcription factors, and cell cycle regulators. Usually, these include changes to the methylation or modification of histones [118].

Research into targeting epigenetic changes in the management of pituitary tumors is limited. The majority of pituitary adenomas have reduced expression of the EGF-containing fibulin-like extracellular matrix protein (*EFEMP1*) gene due to epigenetic silencing, which is reversed in AtT-20 and GH3 cell lines incubated with the DNMT inhibitor zebularine and the HDAC inhibitor trichostatin A (TSA). There was, however, no effect on cell proliferation or apoptotic end points [119]. Bone morphogenetic protein-4 (*BMP-4*) has been proposed as both an oncogene and a tumor suppressor gene in pituitary adenomas Reduced *BMP-4* expression is associated with epigenetic changes, which are reversed in AtT-20 and GH3 cell lines incubated with the epidrugs zebualarine and TSA [120]. Similarly, overexpression of high mobility group A (*HMGA*) genes, a frequent finding in pituitary adenomas, results from epigenetic changes that downregulate microRNA targeting *HMGA* genes. Epidrugs also revert these modifications [121]. The clinical implications of these findings remain to be determined.

## 8. Immune Checkpoint Inhibitor Therapies

**Figure 2:**
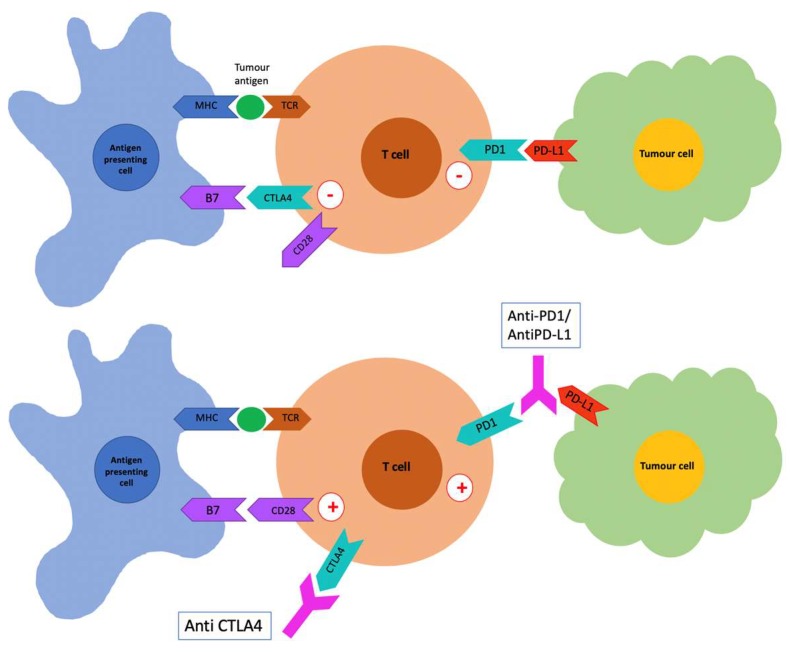
T cell activation requires presentation of tumor antigen to the T cell receptor (TCR) by major histocompatability complex (MHC) on the antigen-presenting cell (APC) and also co-stimulation via B7 on the APC binding to CD28 on the T cell. Cytotoxic T lymphocyte associated protein 4 (CTLA4) is an inhibitory molecule that binds B7 with a higher infinity than CD28 and downregulates the T cell. In addition, PD1 on the T cell binds PD-L1 expressed by tumor cells, which also acts to downregulate the T cell. Immune checkpoint inhibitors, including anti-CTLA4, anti-PD1, and anti-PD-L1 monoclonal antibodies, block the interaction of B7 with CTLA4 and PD1 with PD-L1, eliminating the inhibitory signal and upregulating the T cell.

The significant advances in cancer therapies over the recent years have been based on an increased understanding of the tumor immune microenvironment and development of immunotherapies to target and modulate the immune response. Immune checkpoint inhibitors include anti-CTLA4, anti PD-1 and anti-programmed death ligand 1 (PD-L1) antibodies, which target CTLA4 and PD-1 receptors on the surface of T cells, and PD-L1 expressed on the surface of tumor cells, blocking the subsequent inhibitory signal, and resulting in increased T cell activation and anti-tumor immune response (Figure 2). These agents are effective and have been approved for use in the treatment of multiple malignancies, including melanoma, lung cancer, renal cell carcinoma, squamous cell head and neck cancer, lymphoma, and urothelial carcinoma [122].

Pituitary tumors have been demonstrated to express PD-L1 and CD8+ tumor-infiltrating lymphocytes with higher PD-L1 expression in functioning adenomas and a correlation between PD-L1 expression, hormone levels, and Ki67 [123,124,125]. These findings provide a rationale for exploring the role of immunotherapy in the management of APTs. However, just one case has been reported to date in the literature of a patient with a pituitary carcinoma responding to treatment with the combination checkpoint inhibitor therapy of ipilimumab and nivolumab [25]. The authors presented a case of a 35-year-old female with an aggressive ACTH-secreting pituitary adenoma that initially responded to temozolomide and capecitabine prior to presentation with a liver metastasis. She subsequently received ipilimumab and nivolumab, resulting in a 92% reduction in the volume of the liver metastasis, 59% reduction in the intracranial tumor volume, and normalization of ACTH levels. Interestingly, subsequent genetic analysis of the hepatic metastasis demonstrated development of a hypermutated phenotype (5275 mutations, or 93 mutations/Mb) classic for temozolomide exposure, including an MSH6 mutation. It was postulated that a temozolomide-induced hypermutated tumor may be more sensitive to immune checkpoint inhibitor therapy. It is worth noting that the liver metastasis had low (<1%) PD-L1 expression by immunohistochemistry [25].

This case suggests there may be a role for immune checkpoint inhibitor therapy in the management of APT and PC. The immune microenvironment of pituitary tumors as well as predictors of response in pituitary tumors remain poorly understood and represent new avenues for research.

## 9. Active Clinical Trials

There are four registered active clinical trials examining treatments for patients with APTs. Three trials examining the use of immune checkpoint inhibitor therapies are recruiting, including a phase II trial of Nivolumab Plus Ipilimumab in People with Aggressive Pituitary Tumors (Memorial Sloane Kettering Cancer Centre, United States), Capecitabine and Temozolomide for Treatment of Recurrent Pituitary Adenomas (Weill Cornell Medical College, United States), and Nivolumab and Ipilimumab in Treating Patients with Rare Tumors (University of Alabama et al., United States) [126,127,128]. One study entitled Targeted Therapy with Lapatanib in Patients with Recurrent Pituitary Tumors Resistant to Standard Therapy (Cedars-Sinai Medical Center et al., United States) examined EGFR-targeted therapy and has been completed, although as yet, no results are available [129].

## 10. Conclusions

There is certainly a healthy preclinical research base that provides rationale for exploring a range of targeted therapies, as well as immunotherapy, in the management of APTs. The role of these therapies in APT, however, remains unclear in clinical practice. Unfortunately, rare tumors, such as APTs, are significantly underserved with regard to clinical trial opportunities. Advancement is limited while case reports and occasional case series provide for a slow progression in this field Routine molecular profiling of APTs may both guide treatment decisions and provide justification for access to targeted therapies used routinely in other cancer types. The future may look bright, but for APTs, we are definitely not there yet.

## Figures and Tables

**Table 1:** Published cases of targeted therapies for aggressive pituitary tumors.

	Author	Age	Gender	Subtype	Tumor Molecular Targets	Previous Chemotherapy	Drug	Duration of Treatment (months)	Concurrent Treatment	Outcome
VEGF targeted therapy	Ortiz et al. 2012 [14]	44	Male	Silent ACTH Carcinoma	VEGF immunoreactivity in tumor cell cytoplasm	Temozolomide	Bevacizumab	26	None	SD over 26 months
	O’Riordan et al. 2017 [15]	25	Female	ACTH Carcinoma	None	Temozolomide	Bevacizumab	6	Pasireotide	Reduction in ACTH SD
	Rotman et al. 2019 [16]	51	Male	ACTH	NR	None	Bevacizumab	26	Radiotherapy Temozolomide	Biochemical cure SD >8 years
	Touma et al. 2017 [17]	63	Male	ACTH	NR	None	Bevacizumab	2	Radiotherapy Temozolomide	CR 5 years
	Kurowska et al. 2015 [18]	56	Female	ACTH	NR	Temozolomide	Bevacizumab	NR	None	SD Died from neurosurgical complications
	McCormack et al. 2018 [3]	9	Male	Immuno-negative	NR	Temozolomide	Bevacizumab	4	Temozolomide	PR at 4 months
	McCormack et al. 2018 [3]	46	Male	ACTH	NR	Temozolomide	Bevacizumab	9	None	PR at 9 months
	McCormack et al. 2018 [3]	20	Male	PRL	NR	Temozolomide	Bevacizumab	9	Temozolomide	PD
	McCormack et al. 2018 [3]	55	Male	Immuno-negative	NR	Temozolomide	Bevacizumab	5	None	SD at 5 months
	McCormack et al. 2018 [3]	52	Male	ACTH	NR	Temozolomide	Bevacizumab	2	None	PD
	McCormack et al. 2018 [3]	4	Malr	GH	NR	None	Bevacizumab	21	Temozolomide	PR at 21 months
	Wang et al. 2019 [19]	41	Female	GH	VEGFR-2	None	Apatinib	12	Temozolomide	CR
	McCormack et al. 2018 [3]	NR	NR	NR	NR	NR	Sunitinib	NR	NR	PD
EGFR targeted therapy	Cooper et al. 2019 [20]	NR	Female	PRL	NR	None	Lapatanib	6	None	SD
	Cooper et al. 2019 [20]	NR	Female	PRL	NR	None	Lapatanib	6	None	SD
	Cooper et al. 2019 [20]	NR	Female	PRL	NR	None	Lapatanib	6	None	SD
	Cooper et al. 2019 [20]	NR	Female	PRL	NR	None	Lapatanib	6	None	PR
	Cooper et al. 2019 [20]	NR	Male	PRL	NR	None	Lapatanib	6	None	PD
	Cooper et al. 2019 [20]	NR	Male	PRL	NR	None	Lapatanib	6	None	PD
	McCormack et al. 2018 [3]	NR	NR	NR	NR	NR	Lapatanib	NR	NR	PD
	McCormack et al. 2018 [3]	NR	NR	NR	NR	NR	Lapatanib	NR	NR	PD
	McCormack et al. 2018 [3]	NR	NR	NR	NR	NR	Erlotinib	NR	NR	PD
mTOR inhibition	Zhang et al. 2019 [21]	68	Male	PRL	Increased p-AKT, p-4EBP1 and p-S6	None	Everolimus	16	Cabergoline	PR
	Donovan et al. 2016 [22]	46	Female	ACTH Carcinoma	*STK11* (*F298L*) *NOTCH1* (*R1672H*), *FGFR2*(*P443A*) and *PDGRFRB* (*A713T*) mutation	Capecitabine and temozolomide	Everolimus	7	Capecitabine	SD 5 months
	Jouanneau et al. 2012 [23]	45	Male	ACTH Carcinoma	Activation of AKT1, inactivation of PI3K, overexpression CCND1	Temozolomide	Everolimus	3	Octreotide	PD
	McCormack et al. 2018 [3]	NR	NR	NR	NR	NR	Everolimus	NR	NR	PD
	McCormack et al. 2018 [3]	NR	NR	NR	NR	NR	Everolimus	NR	NR	PD
	McCormack et al. 2018 [3]	NR	NR	NR	NR	NR	Everolimus	NR	NR	PD
CDK4/6 inhibition	Anderson et al. 2018 [24]	71	Female	NR	NR	None	Palbociclib	12	None	PR
ICI therapy	Lin et al. 2018 [25]	35	Female	ACTH	PD-L1 <1%	Capecitabine and temozolomide	Ipilimumab and Nivolumab	4	NR	PR sustained at 6 months

Adrenocorticotroph hormone (ACTH), prolactin (PRL), growth hormone (GH), not reported (NR), stable disease (SD), complete response (CR), partial response (PR), progressive disease (PD), immune checkpoint inhibitor therapy (ICI).

**Table 2:** Overview of targeted therapy for aggressive pituitary tumours and pituitary carcinomas. Therapeutic targets, established drugs, outcomes in other cancers, evidence from pre-clinical studies relating to therapeutic targets in pituitary tumors, and in vitro and in vivo studies of targeted therapy in pituitary tumors.

Target	Drugs	Outcomes in Other Cancers	Results of Pre-clinical Studies	In vitro Therapeutic Studies	In vivo Therapeutic Studies
VEGF targeted therapy	Bevacizumab (VEGFA inhibitor)	Prolongs PFS in metastatic CRC, cervical cancer, non-small cell lung cancer, ovarian cancer, mesothelioma, and metastatic RCC.	Increased VEGF expression in APT compared with non-aggressive pituitary tumors	N/A	Reduced tumor size, prolactin secretion, vascularity and intra-tumoral hemorrhage in prolactin-secreting tumors.
			Octreotide reduces VEGF expression and secretion in GH-secreting adenomas		
	Apatinib (VEGFR-2 inhibitor)	Improved PFS and OS with apatanib in gastric, breast, and lung cancer.	Pasireotide reduces VEGF expression and secretion in NF adenomas		
EGFR targeted therapy	mABs against EGFR	Improved survival in non-small cell lung cancer, metastatic colorectal cancer, head and neck, pancreatic, and breast cancer.	Increased EGFR expression in APT/PCs	Gefitinib suppresses POMC and ACTH in murine and canine corticotroph adenoma cell cultures	Gefitinib attenuates growth and ACTH secretion
	EGFR TKIs (e.g., Gefitinib, Lapatinib)		Increased EGFR expression in corticotroph adenomas	Gefitinib suppresses expression POMC in human corticotroph adenoma cell cultures	
				Lapatinib suppresses prolactin mRNA and secretion and reduces tumor volume in vitro in human prolactinoma cell lines and in vivo in rat models.	
FGF targeted therapy	FGFR TKIs in development	Ongoing investigation in pre-clinical and clinical trials	60% of pituitary adenomas express FGFR-4 isoform, which is not identified in normal pituitary	N/A	N/A
			Increased FGFR-4 in macroadenomas and correlation with Ki67 and tumor invasiveness		
			Increased expression of FGF2 and FGFR-1 in PAs and significantly increased FGFR-1 in invasive tumors		
			Downregulation of FGFR-2 in PAs		
Raf/Mek/ERK pathways	BRAF inhibitors (e.g., Vemurafenib, dabrafenib)	Improved PFS and OS in melanoma	B-Raf mRNA expression and pMEK1/2 and pERK1/2 are increased in pituitary adenomas compared to normal pituitaries	Treatment of murine corticotroph pituitary tumor cells with vemurafenib resulted in a greater reduction of ACTH in the cells expressing the V600E BRAF mutation compared to tumor cells with wild-type BRAF	
			V600E BRAF mutation is identified in corticotroph adenomas		
PI3K/Akt/mTOR	mTOR inhibitors (e.g., Rapamycin and everolimus)	mTOR inhibitors prolong PFS in renal cell carcinoma, neuroendocrine tumors and advanced breast cancer	*PI3KCA* gene amplifications in pituitary tumors	In rat GH3 cell lines, Rapamycin, Everolimus, and PI3K inhibitors decrease cell viability and proliferation.	The pan-PI3K inhibitor Buparlisib reduces tumor volume, prolactin secretion and mitotic index in rat models of prolactin tumors.
				In human GH-secreting PA cell lines, everolimus reduced cell viability in one study but not in another.	
			*PI3KCA* gene mutations are associated with invasive tumors and tumor recurrence	Dual mTOR/P13K inhibitor reduces cell proliferation and promotes cell death in GH3 cells and prolactin secreting cell cultures	
				The combination of everolimus and cabergoline inhibits GH3 cell proliferation and prolactin levels.	
			Akt expression, pAkt, and Akt activity are increased in pituitary tumors compared with normal pituitary tissue	Dual mTOR/PI3K inhibitor reduces cell proliferation and promotes cell death in rat NFPAs	
				The combination of dual PI3K/mTOR inhibition and temozolomide synergistically inhibits tumor growth and reduces GH/PRL levels in pituitary adenoma cell lines and in a mouse GH3 tumor model.	
Notch signaling pathway	Agents targeting Notch are in development	Response demonstrate in Phase 1 and 2 clinical trials in CRC, breast, lung, ovarian and papillary thyroid cancer, anaplastic astrocytoma, sarcoma, glioblastoma multiforme, and melanoma.	Notch 3 receptor and its ligand Jagged1 are increased in NFPAs and PRLs compared with normal pituitary	N/A	N/A
Hedgehog signaling pathway	Vismodegib	Increased OS in metastatic BCC	In PA cell cultures exogenous SHH increased secretion of GH, PRL, and ACTH from their respective tumors	N/A	N/A
			Administration of SHH in corticotroph cell lines exerted anti-proliferative effects		
			Administration of SHH inhibitor increased proliferation in GH3 cell lines		
Cell cycle-targeted therapy	CDK 4/6 inhibitors	Prolong PFS in estrogen receptor positive breast cancer.	Reductions in pRb and p16 or increased expression of cyclin D1 are observed in up to 80% tumors	R-roscovitine (cyclin E/CDK2 inhibitor) reduces cell number, induces cell cycle arrest, induces senescence and reduces ACTH expression and secretion in mouse ACTH-secreting pituitary cells.	R-roscovitine demonstrated a reduction in tumor size and serum and tumor ACTH expression in mice with corticotroph tumors.
			Cyclin D1 is over expressed in aggressive NFPAs.		
			Cyclin E is over expressed and p27 reduced in Cushing’s disease		
			Mutations to p53 are demonstrated in corticotroph adenomas.		
PTTG	N/A	N/A	PTTG is overexpressed in approximately 90% PAs compared with low or no expression in normal pituitary tissue	Overexpression of c-terminal truncated PTTG in rat prolactin- and GH-secreting pituitary tumor GH3 cells suppressed prolactin promotor activity, prolactin mRNA expression and hormonal levels.	Injecting rats with c-terminal-truncated PTTG-transfected GH3 cells resulted in smaller tumors
			PTTG correlates with Ki67 and tumor invasiveness and aggression		
Pituitary Tumor Epigenetics	Zebularine (DNMT)	N/A	Multiple epimutations have been identified in pituitary adenomas	Reversal of epigenetic changes and re-expression of EFEMP1 gene with zebularine and TSA in AtT-20 and GH3 cell lines	N/A
	Trichostatin A (HDAC)			Reversal of epigenetic changes and restored expression of BMP-4 with zebularine and TSA in AtT-20 and GH3 cell lines.	
				Reversal of epigenetic changes and re-expression of HMGA with zebularine and TSA in GH3 cell lines.	
ICI therapy	Anti PD-1, anti PD-L1, Anti CTLA4 antibodies	Effective and approved for use in the treatment of melanoma, lung cancer, RCC, head and neck SCC, lymphoma, and urothelial carcinoma	Pituitary tumors express PD-L1 and CD8+ tumor infiltrating lymphocytes with higher PD-L1 expression in functioning adenomas and a correlation between PD-L1 expression and hormonal levels and Ki67	N/A	N/A

Aggressive pituitary tumor (APT), pituitary carcinoma (PC), vascular endothelial growth factor (VEGF), vascular endothelial growth factor receptor (VEGFR) progression free survival (PFS), overall survival (OS), colorectal cancer (CRC), renal cell carcinoma (RCC), growth hormone (GH), epidermal growth factor receptor (EGFR), monoclonal antibodies (mABs), tyrosine kinase inhibitors (TKIs), fibroblast growth factor (FGF), fibroblast growth factor receptor (FGFR), pituitary adenoma (PA), non-functioning pituitary adenomas (NFPAs), basal cell carcinoma (BCC), sonic hedgehog (SHH), pituitary tumor transforming gene (PTTG), DNA methyltransferase (DNMT), histone deacetylase (HDAC), EGF containing fibulin-like extracellular matrix protein (EFEMP1), high mobility group A (HMGA), immune checkpoint inhibitor (ICI), programmed cell death protein 1 (PD-1), programmed death ligand 1 (PD-L1), cytotoxic T lymphocyte associated protein 4 (CTLA4).
